# FISH landmarks reflecting meiotic recombination and structural alterations of chromosomes in wheat (*Triticum aestivum* L.)

**DOI:** 10.1186/s12870-021-02947-1

**Published:** 2021-04-06

**Authors:** Yang Zou, Linrong Wan, Jie Luo, Zongxiang Tang, Shulan Fu

**Affiliations:** 1 College of Agronomy, Sichuan Agricultural University, Wenjiang, 611130 Sichuan China; 2 Institute of Ecological Agriculture, Sichuan Agricultural University, Wenjiang, 611130 Sichuan China; 3 Provincial Key Laboratory for Plant Genetics and Breeding, Wenjiang, 611130 Sichuan China

**Keywords:** Wheat, Tandem repeats, Meiotic recombination, Chromosome condensation

## Abstract

**Background:**

DNA sequence composition affects meiotic recombination. However, the correlation between tandem repeat composition and meiotic recombination in common wheat (*Triticum aestivum* L.) is unclear.

**Results:**

Non-denaturing fluorescent in situ hybridization (ND-FISH) with oligonucleotide (oligo) probes derived from tandem repeats and single-copy FISH were used to investigate recombination in three kinds of the long arm of wheat 5A chromosome (5AL). 5AL^535–18/275^ arm carries the tandem repeats pTa-535, Oligo-18, and pTa-275, 5AL^119.2–18/275^ arm carries the tandem repeats pSc119.2, Oligo-18 and pTa-275, and 5AL^119.2^ arm carries the tandem repeats pSc119.2. In the progeny of 5AL^535–18/275^ × 5AL^119.2^, double recombination occurred between pSc119.2 and pTa-535 clusters (119–535 interval), and between pTa-535 and Oligo-18/pTa-275 clusters (535–18 interval). The recombination rate in the 119–535 interval in the progeny of 5AL^535–18/275^ × 5AL^119.2–18/275^ was higher than that in the progeny of 5AL^535–18/275^ × 5AL^119.2^. Recombination in the 119–535 interval produced 5AL^119 + 535^ segments with pTa-535 and pSc119.2 tandem repeats and 5AL^No^ segments without these repeats. The 5AL^119 + 535^ and 5AL^No^ segments were localized between the signal sites of the single-copy probes SC5A-479 and SC5A-527. The segment between SC5A-479 and SC5A-527 in the metaphase 5AL^No^ was significantly longer than that in the metaphase 5AL^119 + 535^.

**Conclusion:**

The structural variations caused by tandem repeats might be one of the factors affecting meiotic recombination in wheat. Meiotic recombination aggregated two kinds of tandemly repeated clusters into the same chromosome, making the metaphase chromosome more condensed. To conclude, our study provides a robust tool to measure meiotic recombination and select parents for wheat breeding programs.

**Supplementary Information:**

The online version contains supplementary material available at 10.1186/s12870-021-02947-1.

## Background

Increasing the chromosomal recombination rate can accelerate new cultivars’ development in common wheat (*Triticum aestivum* L.). Researchers have extensively studied the molecular mechanisms of meiotic recombination in plants and reported many genes involved in the process [1], which provide an opportunity to manipulate the mechanisms for crop improvement [2]. Mutation in the anticrossover gene *FANCM* (Fanconi anemia complementation group M) resulted in a two-fold increase in meiotic recombination in hybrid rice and pea [3]. However, the *fancm* mutation has almost no effect on recombination in hybrid *Arabidopsis* but resulted in a three-fold increase in inbreds [3–5]. Combining the anticrossover mutants *recq4* (RecQ like helicase 4) and *figl1* (FIDGETIN-like-1) resulted in a 7.8-fold increase in crossover frequency, while the *fancm*, *recq4* and *figl1* triple mutant displayed less recombination [5]. These findings indicated that factors other than genes help control meiotic recombination. Both genes and chromatin structure control the recombination rate. In humans and animals, the recombination rate is related to cytogenetic structures that were displayed by staining intensity of G bands and sequence compositions including repetitive elements, GC content, CpG density and poly(A)/poly(T) stretches [6, 7]. In *Arabidopsis thaliana*, A-rich, CCN-repeat and CTT-repeat motifs are enriched in the crossover regions [8]. Reduced DNA methylation at the CG sites increases recombination rate in the euchromatic, but not in the pericentric heterochromatin regions [9]. Heterochromatin plays a role in meiotic recombination. It represses centromeric meiotic recombination in fission yeast [10]. The role of pericentric heterochromatin in suppressing meiotic recombination has been widely studied in eukaryotes [11]. The disruption of H3K9me2 and non-CG DNA methylation pathways via gene mutations increased pericentromeric crossovers in hybrid and inbred *Arabidopsis* [12]. Meanwhile, in mice, a tandem array of mo-2 minisatellite conferred higher-order structures crucial for recombination in the pseudoautosomal region of sex chromosomes [13]. Therefore, the effect of tandem repeats on chromosomal recombination should be investigated. Common wheat can be used as a reference model to study the role of tandem repeats in chromosomal recombination. Previous studies have indicated a correlation between the wheat chromosomal crossover and gene-rich regions [14–18]. However, different aspects of recombination should be considered for common wheat because of allopolyploidy and repetitive sequences [19].

Some new tandem repeats were discovered from common wheat [20, 21]. In our previous work, oligonucleotide (oligo) probes derived from these tandem repeats displayed large structural variations in the 5AL arms of common wheat [22, 23]. However, the correlation between the composition of tandem repeats and the rate of recombination in wheat is still unclear. This study investigates the effect of genomic structural variations reflected by tandem repeats on recombination frequency in the wheat chromosome 5A.

## Results

### FISH karyotypes of 5A chromosomes

In wheat, the 5A chromosomes can be distinguished from the other chromosomes based on the signal patterns of Oligo-713, Oligo-pSc119.2–1, and Oligo-pTa535–1 [22, 24]. The Oligo-pSc119.2–1 signals on the telomeric region of 5BS (the short arm of 5B chromosome) are stronger than that on 5AS (the short arm of 5A chromosome), and the signal patterns of Oligo-pTa535–1 on 5D chromosomes are different from that on 5A chromosomes [22, 24]. However, when Oligo-pSc119.2–1 signals occur on both 5AS and 5AL, it is challenging to distinguish 5A and 2B chromosomes. Then, the 5A chromosomes are identified based on the submetacentric feature. The pericentromeric region of 7AS (the short arm of the 7A chromosome) and 7DS (the short arm of the 7D chromosome) contains Oligo-713 signals, however, the 7A and 7D chromosomes can be distinguished from 5A chromosomes based on the signal patterns of Oligo-pSc119.2–1 and Oligo-pTa535–1 [22].

**Fig. 1:**
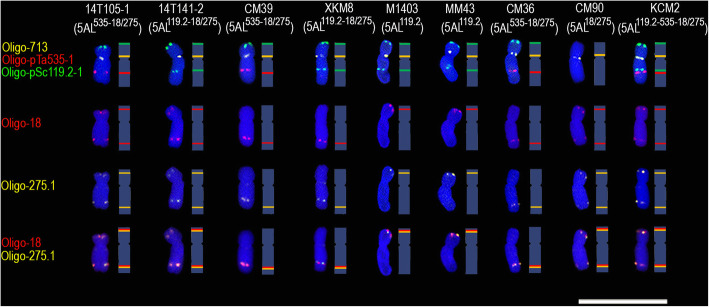
Signal patterns of five oligo probes on the metaphase 5A chromosomes of nine wheat cultivars/lines. The schematic representation of each chromosome is shown. Scale bar: 50 μm

The non-denaturing fluorescence in situ hybridization (ND-FISH) of this study indicated that the signals of Oligo-713 occurred on the pericentromeric region of 5AS arms of all the nine wheat cultivars/lines (Fig. 1). The probe Oligo-pSc119.2–1 produced signals on 5AS arms of eight wheat cultivars/lines, except for CM90, and on 5AL arms of 14 T141–2, XKM8, M1403, and MM43 (Fig. 1). The signals of Oligo-pTa535–1 occurred on 5AL arms of 14 T105–1, CM36, and CM39 (Fig. 1). Both Oligo-pSc119.2–1 and Oligo-pTa535–1 probes produced signals on 5AL arms of KCM2 (Fig. 1). The signals of Oligo-275.1 and Oligo-18 occurred on both 5AS and 5AL arms of 14 T105–1, 14 T141–2, CM90, and KCM2 (Fig. 1), and 5AL arms of CM39, XKM8 and CM36 (Fig. 1). Although Oligo-275.1 and Oligo-18 signals appeared on 5AS arms of M1403 and MM43, they did not occur on 5AL arms of the two cultivars (Fig. 1). Therefore, we named the 5AL arms as follows: 14 T105–1, CM39, and CM36 as 5AL^535–18/275^; 14 T141–2 and XKM8 as 5AL^119.2–18/275^; M1403 and MM43 as 5AL^119.2^; CM90 as 5AL^18/275^; and KCM2 as 5AL^119.2–535-18/275^ (Fig. 1).

### FISH karyotypes and recombination rate of the F_2_ generation

**Fig. 2:**
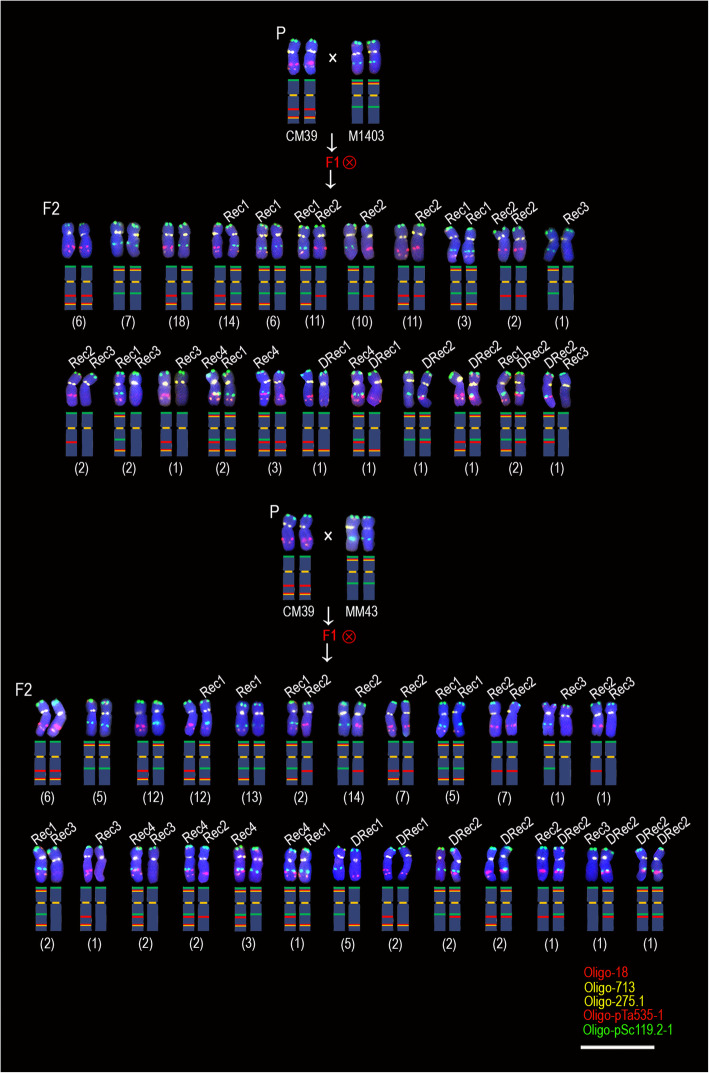
Combination types of 5A chromosomes in the F_2_ progeny derived from CM39 × M1403 and CM39 × MM43. ‘P’ indicates parental plants. ‘F1’ indicates F_1_ generation. ‘Ⓧ’ indicates selfing. ‘F2’ indicates F_2_ generation. ‘Rec1’ and ‘Rec2’ indicate the 5AL arms formed by recombination in the 535–18 interval. ‘Rec3’ and ‘Rec4’ indicate the 5AL arms formed by recombination in the 119–535 interval. ‘DRec1’ and ‘DRec2’ indicate the 5AL arms formed by recombination in both 535–18 and 119–535 intervals. The number in parentheses indicates the number of seeds that contain the corresponding combination types of 5A chromosomes. The schematic representation of each chromosome is shown. Scale bar: 50 μm

**Fig. 3:**
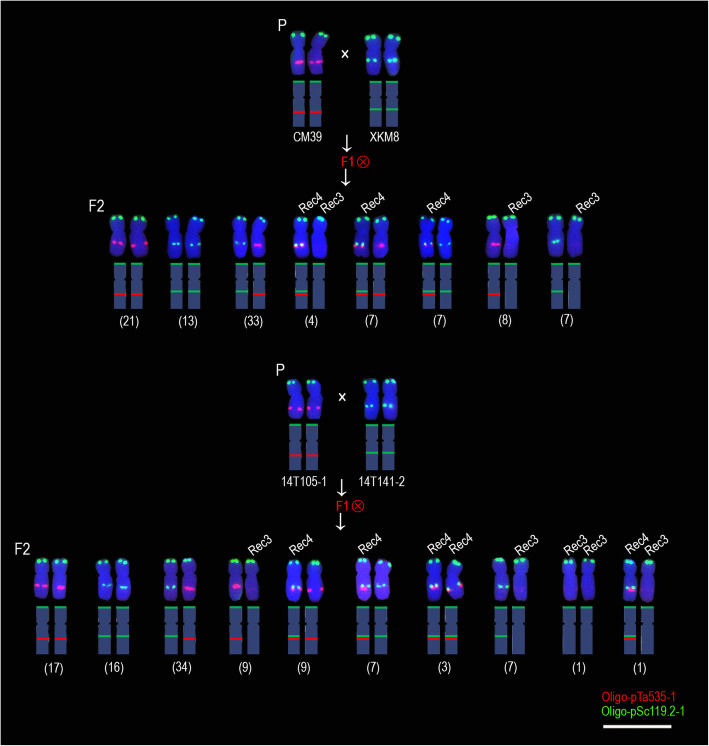
Combination types of 5A chromosomes in the F_2_ progeny derived from CM39 × XKM8 and 14 T105–1 × 14 T141–2. ‘P’ indicates parental plants. ‘F1’ indicates F_1_ generation. ‘Ⓧ’ indicates selfing. ‘F2’ indicates F_2_ generation. ‘Rec1’ and ‘Rec2’ indicate the 5AL arms formed by recombination in the 535–18 interval. ‘Rec3’ and ‘Rec4’ indicate the 5AL arms formed by recombination in the 119–535 interval. ‘DRec1’ and ‘DRec2’ indicate the 5AL arms formed by recombination in both 535–18 and 119–535 intervals. The number in parentheses indicates the number of seeds that contain the corresponding combination types of 5A chromosomes. The schematic representation of each chromosome is shown. Scale bar: 50 μm

A total of 420 F_2_ generation seeds (840 5A chromosomes) derived from the CM39 × M1403, CM39 × MM43, CM39 × XKM8, and 14 T105–1 × 14 T141–2 hybrid combinations were analyzed to determine the recombination rate of 5AL arms. These included 106 seeds (212 5A chromosomes) derived from CM39 × M1403 (5AL^535–18/275^ × 5AL^119.2^), 110 (220 5A chromosomes) from CM39 × MM43 (5AL^535–18/275^ × 5AL^119.2^), 100 (200 5A chromosomes) from CM39 × XKM8 (5AL^535–18/275^ × 5AL^119.2–18/275^) and 104 (208 5A chromosomes) from 14 T105–1 × 14 T141–2 (5AL^535–18/275^ × 5AL^119.2–18/275^). For the progeny derived from CM39 × M1403 and CM39 × MM43, two recombination intervals were detected (Fig. 2 and Additional files 1, 2). One recombination occurred between the signal sites of Oligo-pSc119.2–1 and Oligo-pTa535–1, and this region was named 119–535 interval. The other recombination occurred between the signal sites of Oligo-pTa535–1 and Oligo-18/Oligo-275.1, and this region was named 535–18 interval (Fig. 2 and Additional files 1, 2). For the progeny of CM39 × XKM8 and 14 T105–1 × 14 T141–2, only the recombination occurred in the 119–535 interval was observed because the Oligo-275.1 and Oligo-18 signals were detected on both 5AL^535–18/275^ and 5AL^119.2–18/275^ arms of (Fig. 3 and Additional file 3). The 106 seeds of CM39 × M1403 had 22 combination types of 5A chromosomes (Fig. 2), and the recombination occurred in 119–535 and 535–18 intervals on 20 and 88 5A chromosomes, respectively, including the seven chromosomes with double recombination (Fig. 2). The 110 seeds of CM39 × MM43 had 25 combination types of 5A chromosomes (Fig. 2). Of the 220 5A chromosomes, recombination occurred in 119–535 and 535–18 intervals on 31 and 96 5A chromosomes, respectively, including the 15 chromosomes with double recombination (Fig. 2). Only eight combination types of 5A chromosomes were observed among the 100 seeds (200 chromosomes) derived from CM39 × XKM8, and recombination in the 119–535 interval was observed on 37 5A chromosomes (Fig. 3). The signals of Oligo-713, Oligo-275.1, and Oligo-18 were not shown on these 5A chromosomes because they could not reflect recombination in the 535–18 interval. Similarly, only 10 combination types of 5A chromosomes were observed among the 104 seeds (208 5A chromosomes) derived from 14 T105–1 × 14 T141–2, and recombination in the 119–535 interval occurred on 42 5A chromosomes (Fig. 3).

**Fig. 4:**
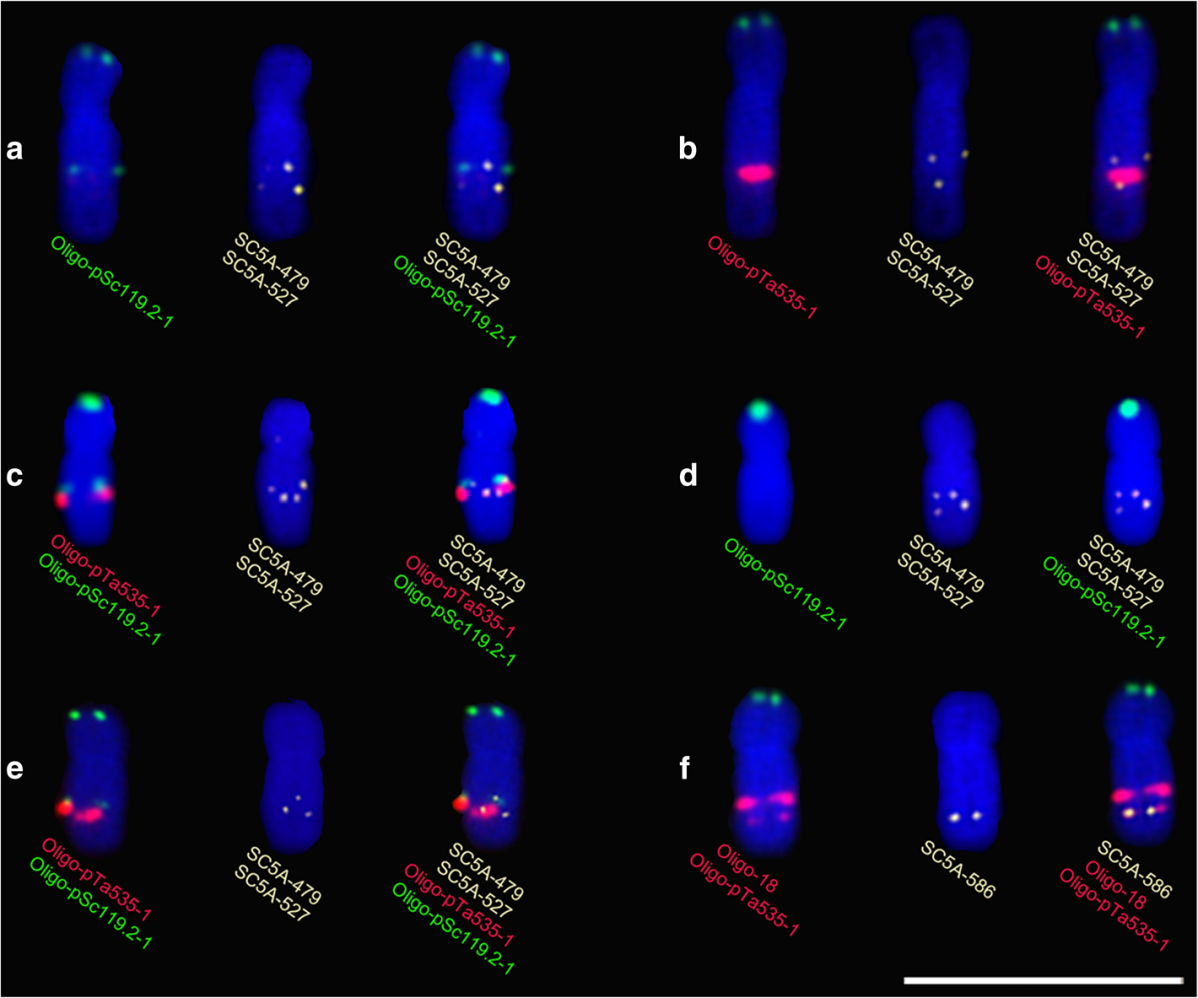
Position of the signal sites of single-copy probes and oligo probes on 5AL arms. **a** 5A chromosome derived from MM43 showing the 5AL^119^ segment corresponding to the 119–535 interval between SC5A-479 and SC5A-527 sites. **b** 5A chromosome derived from CM39 showing the 5AL^535^ segment corresponding to the 119–535 interval between SC5A-479 and SC5A-527 sites. **c** 5A chromosome formed by recombination in the 119–535 interval showing the 5AL^119 + 535^ segment between SC5A-479 and SC5A-527 sites. **d** 5A chromosome formed by recombination in the 119–535 interval showing the 5AL^No^ segment between SC5A-479 and SC5A-527 sites. **e** 5A chromosome of KCM2 containing the 5AL^119 + 535^ segment between SC5A-479 and SC5A-527 sites. **f** 5A chromosome of CM39 showing the close signal sites of SC5A-586 and Oligo-18. Scale bar: 50 μm

**Fig. 5:**
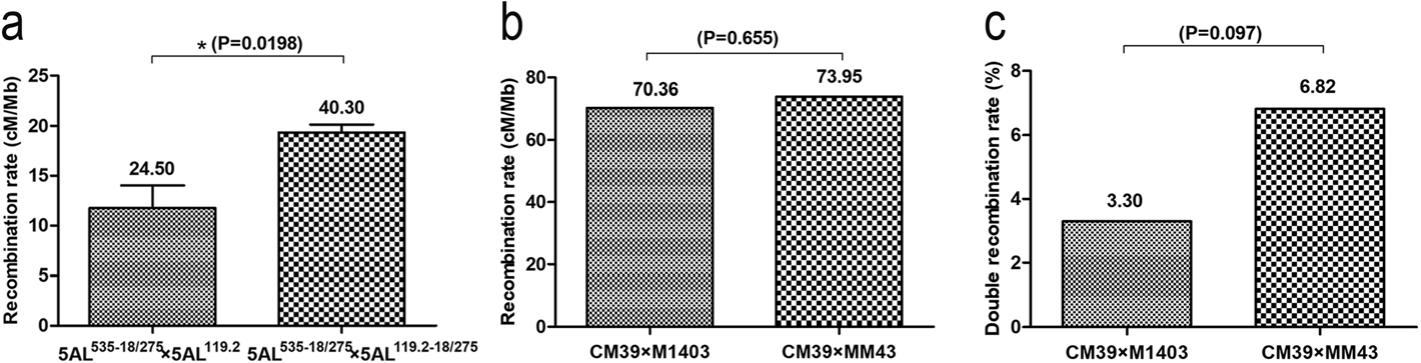
Statistical analysis of recombination rate. **a** Comparison of recombination rate in the 119–535 interval. **b** Comparison of recombination rate in the 535–18 interval. **c** Comparison of double recombination rate in the 119–535 and 535–18 intervals. *: *p* < 0.05

Sequential single-copy FISH and ND-FISH assays indicated that the signal sites of Oligo-pSc119.2–1 and SC5A-479 were close each other (Fig. 4a, c and Additional file 4c, f) and that of Oligo-pTa535–1 and SC5A-527 were close each other (Fig. 4b, c and Additional file 4c, f). The signal sites of SC5A-479 and SC5A-527 on 5AL^119.2–535-18/275^ arms of KCM2 confirmed this observation (Fig. 4e and Additional file 4i). Additionally, the signals of Oligo-18 and SC5A-586 were close (Fig. 4f and Additional file 4 l) (The details of obtaining the three single copy probes are described in Methods section.). The 119–535 and 535–18 intervals were determined as 48 Mb and 59 Mb long, respectively, based on the physical distance of the single-copy probes SC5A-479, SC5A-527, and SC5A-586. The recombination rate of the 5AL arms in the four kinds of F_2_ populations is shown in Table 1. The recombination rate in 119–535 interval in the progeny derived from CM39 × XKM8 (5AL^535–18/275^ × 5AL^119.2–18/275^) and 14 T105–1 × 14 T141–2 (5AL^535–18/275^ × 5AL^119.2–18/275^) was higher than that in the progeny derived from the other two combinations (5AL^535–18/275^ × 5AL^119.2^) (Table 1), and a significant difference was observed between the progeny of 5AL^535–18/275^ × 5AL^119.2–18/275^ and 5AL^535–18/275^ × 5AL^119.2^ (Fig. 5a). However, no significant difference was observed for the recombination rate in 535–18 interval between the progeny of CM39 × M1403 (5AL^535–18/275^ × 5AL^119.2^) and CM39 × MM43 (5AL^535–18/275^ × 5AL^119.2^) (Fig. 5b). Our analysis indicated that crossover interference existed between 119 and 535 and 535–18 intervals (Table 1), however, no significant difference was observed for the double recombination rate between the progeny of CM39 × M1403 (5AL^535–18/275^ × 5AL^119.2^) and CM39 × MM43 (5AL^535–18/275^ × 5AL^119.2^) (Fig. 5c) (The details of the calculating of recombination and interference are described in Methods section.).

**Table 1:** Recombination rate of 5AL arm in F_2_ populations derived from different combinations

Hybrid combination	CM39 × M1403	CM39 × MM43	CM39 × XKM8	14 T105–1 × 14 T141–2
119–535 cM	9.43	14.09	18.50	20.19
535–18 cM	41.51	43.63	–	–
Recombination rate in 119–535 interval (cM/Mb)	19.65	29.35	38.54	42.06
Recombination rate in 535–18 interval (cM/Mb)	70.36	73.95	–	–
Obs DCO	3.30	6.82	–	–
Exp DCO	8.30	13.52	–	–
CoC	0.378	0.544	–	–
Interference	0.622	0.456	–	–

“-” means iterms cannot be determined

### Relative length of the metaphase chromosome segment between SC5A-479 and SC5A-527

**Fig. 6:**
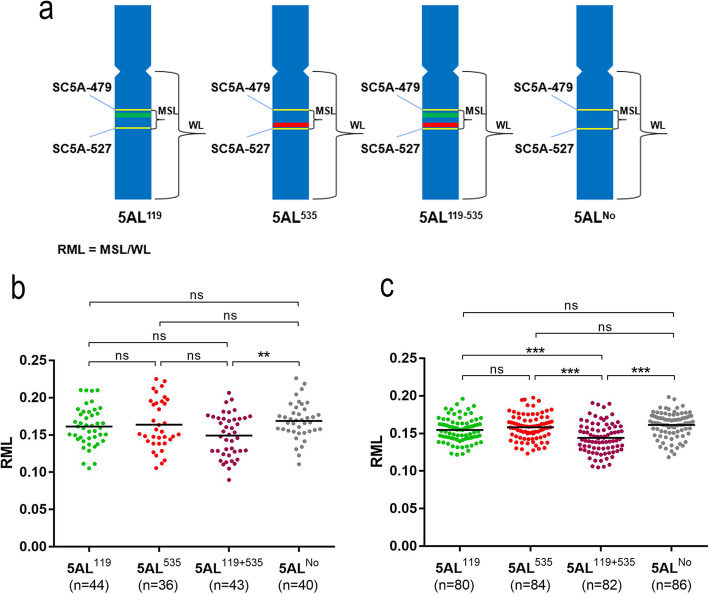
Comparison of 5AL^119^, 5AL^535^, 5AL^119 + 535^, and 5AL^No^ segments. **a** Model for the measurement of the segments on metaphase 5AL arms. Yellow bands indicate the signals of single-copy probes SC5A-479 and SC5A-527. Green bands indicate the signals of Oligo-pSc119.2–1. Red bands indicate the signals of Oligo-pTa535–1. ‘WL’ means the whole length of the 5AL arm. ‘MSL’ means the length of metaphase 5AL segment between SC5A-479 and SC5A-527 probes. **b** Comparison of relative metaphase length (RML) among the four kinds of segments in the four F_2_ generations. **c** Comparison of RML among the four kinds of segments in the four wheat cultivars. Each dot represents the RML of an individual 5AL segment. The bars among the dots represent the mean values of RML. The number of 5AL segments (n) measured for each kind of segment is shown in parentheses. ns: no significant difference, **: *p* < 0.01, ***: *p* < 0.001

Figures 2 and 3 show that the recombination in the 119–535 interval formed the recombination types Rec3, Rec4 and DRec2. The signals of both Oligo-pSc119.2–1 and Oligo-pTa535–1 occurred on type Rec4 5AL arms that disappeared from type Rec3 5AL arms (Figs. 2, 3 and Additional files 1, 2, 3). The 119–535 interval corresponds to the segment between the signal sites of SC5A-479 and SC5A-527 on metaphase 5AL arms (Fig. 4 and Additional file 4). We named the segment between SC5A-479 and SC5A-527 on 5AL^119.2^, 5AL^535–18/275^, Rec3, and Rec4 5AL arms as 5AL^119^, 5AL^535^, 5AL^No^, and 5AL^119 + 535^, respectively. The relative metaphase length (RML) of the four kinds of metaphase 5AL segments (5AL^119^, 5AL^535^, 5AL^119 + 535^, and 5AL^No^) was measured (Fig. 6a). From the four types of F_2_ generations, a total of 44, 36, 43 and 40 segments of 5AL^119^, 5AL^535^, 5AL^119 + 535^ and 5AL^No^, respectively, were successfully measured (Fig. 6b). The 5AL^No^ segment was significantly longer than 5AL^119 + 535^ (Fig. 6b). Further, the 5AL^119^, 5AL^535^, 5AL^119 + 535^, and 5AL^No^ segments (Fig. 1) in the wheat cultivars XKM8, CM36, KCM2 and CM90 were also measured to confirm this (Fig. 6c). A total of 80, 84, 82, and 86 5AL^119^, 5AL^535^, 5AL^119 + 535^, and 5AL^No^ segments were obtained and measured from the four wheat cultivars (Fig. 6c). The 5AL^119^, 5AL^535^, and 5AL^No^ segments were significant longer than 5AL^119 + 535^ (Fig. 6c).

## Discussion

### Correlation between tandem repeats and meiotic recombination

The four kinds of tandem repeats pSc119.2, pTa-535, pTa-275 and Oligo-18 displayed apparent structural variations in the 5AL arms of wheat. In this study, the cross combinations CM39 × XKM8, CM39 × M1403 and CM39 × MM43 have the same parent CM39. However, the recombination rate in the 119–535 interval was lower in the progeny derived from CM39 × M1403 (5AL^535–18/275^ × 5AL^119.2^) and CM39 × MM43 (5AL^535–18/275^ × 5AL^119.2^) than that in the progeny derived from CM39 × XKM8 (5AL^535–18/275^ × 5AL^119.2–18/275^). Besides, the 5AL arms of M1403 and MM43 showed ND-FISH signal patterns different from that of XKM8. It was speculated that recombination rate variation in the 119–535 interval is consistent with the ND-FISH signal patterns on 5AL arms, supported by the progeny derived from 14 T105–1 × 14 T141–2 (5AL^535–18/275^ × 5AL^119.2–18/275^). Therefore, a correlation between the composition of tandem repeats in 5AL arms and meiotic recombination might existed.

Researchers have observed a higher rate of motifs of DNA transposons in the recombination intervals. In potato, the *Stowaway* family of miniature inverted-repeat transposable elements spanned the crossover regions [25]. Darrier et al. observed a higher frequency of a DNA motif specific to the *TIR*-*Mariner* DNA transposon in common wheat recombinant intervals [16]. These studies found differences in DNA transposon composition between recombination and non-recombination hotspots along the same chromosome, a phenomenon observed in diverse populations [16, 25]. Putative sequence divergence or insertions/deletions in wheat led to significant differences in crossover frequency along 3B chromosomes between two different F_2_ segregating populations [15]. Meanwhile, a nested association mapping (NAM) population used to map the QTL affecting the crossover distribution and frequency indicated a lower recombination rate in the regions with more single nucleotide polymorphisms (SNPs) than with fewer SNPs in wheat [17]. Besides, similar crossover patterns were detected in populations derived from closely related parents [18]. Even in the mutants of anticrossover genes, recombination was prevented in regions with the highest sequence divergence, displayed by SNP [3]. In *Arabidopsis*, the *msh2* (MutS-related heterodimers) mutant displayed significantly reduced SNP enrichment around crossovers compared with the wild type [26]. These studies indicated that SNP polymorphisms and indels are important factors affecting meiotic recombination. Although a significant difference was observed in the recombination rate in the 119–535 interval between the progeny derived from 5AL^535–18/275^ × 5AL^119.2^ and 5AL^535–18/275^ × 5AL^119.2–18/275^ (Fig. 5a), other sequence differences among the 5AL arms, especially in 119–535 and 535–18 intervals, that result in different genetic distances cannot be ignored. In addition to the tandem repeats, these sequence components may also affect the recombination rate between the different 5AL arms. Meanwhile, the recombination rate in the 119–535 interval in the progeny derived from 5AL^535–18/275^ × 5AL^119.2–18/275^ was higher than that in the progeny derived from 5AL^535–18/275^ × 5AL^119.2^. Therefore, the effects of tandem repeats on meiotic recombination should be further investigated.

### Effects of juxtaposed heterozygous and homozygous regions on recombination

Although it cannot be concluded that the composition of tandem repeats alone resulted in a comparatively low recombination rate in the 119–535 interval in the progeny of CM39 × M1403 (5AL^535–18/275^ × 5AL^119.2^) and CM39 × MM43 (5AL^535–18/275^ × 5AL^119.2^), the variations in the recombination rate in this interval are consistent with the structural differences in the 5AL arms. Different ND-FISH patterns occurred at the pSc119.2 and pTa-535 sites between 5AL^535–18/275^ and 5AL^119.2–18/275^. However, the different ND-FISH patterns were observed between 5AL^535–18/275^ and 5AL^119.2^ at the pSc119.2, pTa-535, and Oligo-18/pTa-275 sites. In *Arabidopsis*, heterozygous regions increase crossover in juxtaposed megabase homozygous and heterozygous regions, and heterozygosity can increase crossover interference [27]. Thus, our findings, together with these previous reports, indicate the need to investigate the effects of juxtaposition of heterozygous and homozygous intervals on meiotic recombination.

### Effects of tandem repeats on metaphase chromosome condensation

In this study, recombination formed the 5AL^119 + 535^ and 5AL^No^ metaphase segments. A significant difference in length was observed only between 5AL^119 + 535^ and 5AL^No^ segments derived from the F_2_ populations; 5AL^119 + 535^ of KCM2 was significantly shorter than that of XKM8 (5AL^119^), CM36 (5AL^535^), and CM90 (5AL^No^) (Fig. 6b, c). These observations indicate other sequence differences in the 119–535 interval of the 5AL arms, affecting the length of 5AL^119^, 5AL^535^, 5AL^119 + 535^ and 5AL^No^ segments. However, for both F_2_ populations and wheat cultivars, the 5AL^No^ segments were significantly longer than the 5AL^119 + 535^ segments. These findings indicate that the metaphase chromosome segments containing two tandemly repeated clusters close to each other in the same chromosome will be more condensed. Conversely, the metaphase chromosome segments with fewer tandem repeats will be more relaxed. Moreover, the cellSens Dimension software-based method used in this study to measure the length is reliable. Tandemly repeated satellite DNA sequences can drive population and species divergence by inducing alterations in heterochromatin and/or centromere [28]. Satellite DNA sequences are the major heterochromatin components that play an essential role in heterochromatin formation and regulation [29]. In mouse, the heterochromatin alters the loop size of the chromatin of mitotic chromosome [30]. Therefore, the 5AL^119 + 535^ segment of this study might be highly heterochromatinized by the aggregating pSc119.2 and pTa-535 making the segment more condensed.

### Application of ND-FISH in wheat breeding programs

Understanding the factors affecting the variations in recombination rate and recombination point is important in wheat breeding programs. Generally, crossover frequency decreases from telomere to centromere in eukaryotes [31], including wheat [15, 16, 32]. Mutations in the anticrossover genes do not increase the recombination in regions close to centromere [3]. However, increasing the interstitial recombination rate reduces deleterious genetic load, which will benefit crop improvement [17]. Therefore, we should understand the mechanisms and methods to increase the recombination rate in the proximal regions [3]. The differences in recombination rate detected in this study reflect differences in sequence composition in 119–535 and 535–18 intervals derived from different crosses. ND-FISH assay displays the differences in tandem repeat composition in wheat chromosomes and can be used to predict the recombination rate.

Earlier, Derrier et al. used chromosome 3B pseudomolecule and high-throughput SNP detection to map 252 crossover events at intervals of < 26 kb, a high-resolution crossover location in common wheat [16]. Although high-throughput sequencing and DNA markers have great advantages in studying chromosomal recombination [15–18, 32], large number of individual recombinants need to be sequenced. Cytological markers have advantages of visualization and intuition in studying chromosomal recombination [33–35]. ND-FISH technology based on oligo probes can be used to establish abundant FISH karyotypes of wheat chromosomes conveniently [21, 36], and oligo probes derived from tandem repeats can display structural variations in wheat chromosomes [22, 23, 36, 37]. Besides, chromosomal recombination indicated by FISH karyotypes is visual comprehend and can be used conveniently in wheat breeding programs [34, 35]. Therefore, high-throughput sequencing and DNA markers should be combined with FISH karyotypes to study the wheat chromosomal recombination rules.

## Conclusion

The present study confirms the effects of tandem repeats on meiotic recombination in wheat, however, studies should be carried out to identify other factors determining the recombination rate in 5AL arms. Our study provides a robust visual tool based on ND-FISH to measure meiotic recombination and crossover interference in wheat.

## Methods

### Plant materials

Four wheat cultivars Chuanmai 39 (CM39), Xikemai 8 (XKM8), Mian 1403 (M1403) and Mianmai 43 (MM43), and two wheat lines 14 T105–1 and 14 T141–2 were used as parents for hybridization. Four cross combinations, CM39 × M1403, CM39 × MM43, CM39 × XKM8, and 14 T105–1 × 14 T141–2, were carried out, and four F_2_ generations were obtained by selfing. The wheat cultivars Chuanmai 36 (CM36), Chuanmai 90 (CM90), and Kechengmai 2 (KCM2) were used to measure the distance between the FISH signals of the single-copy probes SC5A-479 and SC5A-527 on the 5AL arms at metaphase.

### Non-denaturing FISH (ND-FISH)

Oligo-pSc119.2–1, Oligo-pTa535–1 [24], Oligo-713, Oligo-275.1 [22], and Oligo-18 [21] were used as oligo probes for ND-FISH. The information on these oligo probes is listed in Additional file 5. These oligo probes can replace their original sequences’ roles in identifying wheat chromosomes [22, 24]. The sequence of the Oligo-18 probe is identical to the original one, both are 18 bp long [21]. The metaphase chromosomes were prepared from the root tips according to the method described by Han et al. [38]. ND-FISH was performed according to the method described by Tang et al. [21]. An epifluorescence microscope (BX51, Olympus Corporation, Tokyo, Japan) with cellSens Dimension software (Olympus Corporation, Tokyo, Japan) was used to capture images.

### Single-copy FISH

The Tandem Repeat Finder (TRF, Version 4.09) [39] program was used to filter the tandem repeats in the 5A chromosome (IWGSC RefSeq Version 2.0). An in-house R package was then used to filter other repeated sequences, including retrotransposon and transposon elements. The remaining sequences were used as the query to align with the full-length sequence of chromosome 5A of the bread wheat variety Chinese Spring (IWGSC RefSeq Version 2.0) using the in-house R package. The query sequences aligned to themselves at a single site were kept as preparatory probes. The sequences of Oligo-pSc119.2–1, Oligo-pTa535–1 [24], and Oligo-18 [21] were also used as the query to align with the full-length sequence of 5A chromosome using BLAST in the B2DSC web server (http://mcgb.uestc.edu.cn/b2dsc) [40]. The sequences of Oligo-pTa535–1 and Oligo-pSc119.2–1 hit their targets with high copy numbers at 431–432 Mbp and 507–508 Mbp sites on the 5AL arm, respectively. These were used to narrow down the options for single copy probes. Finally, two single-copy sequences, SC5A-479 (479272790–479,275,822 bp) and SC5A-527 (527288389–527,289,202 bp), were selected as probes. The sequence of Oligo-18 hit the target with a high copy number at one site (584–585 Mbp) on the 5AL arm. Then a single copy sequence SC5A-586 (586379936–586,380,903 bp) was selected as the probe. The primer pairs of SC5A-479 (5’TCGTTGACTAGAAAGACGTG TGT3’, 5’ACGCCTGTGTTAAGTTAAGTGAC3’), SC5A-527 (5’TGCGTACATAGGGTGAGTGTATG3’, 5’GGCCTCTGGAAGAACGTTTTAT3’), and SC5A-586 (5’TTGCTCGTGTCCACCATTGA3’, 5’TGTGGAATACTTACCGCGCA3’) were used to amplify the three single-copy probe sequences. The target sequences were cloned into the TSINGKE pClone007 vector (TSINGKE, Chengdu, China) and labeled with Texas-Red-5-dUTP (PerkinElmer, USA) according to the method described by Han et al. [38]. The root tip metaphase chromosomes were prepared, and the hybridization was performed as described by Han et al. [38] with slight modifications. The probe mixture contained 5–6 ng/μL of each probe and 1 × ENZO buffer (ENZO Life Science Inc. USA). Slides were washed in 2 × SSC buffer containing 0.1% NP-40 detergent (Solarbio Life Sciences Ltd. China) for 3–5 min at 45–50 °C. An epifluorescence microscope (BX51, Olympus Corporation, Tokyo, Japan) with cellSens Dimension software (Olympus Corporation, Tokyo, Japan) was used to capture the images.

### Calculation of recombination rate and crossover interference

Recombination was determined based on the FISH patterns of the probes Oligo-pSc119.2–1, Oligo-pTa535–1, Oligo-275.1, and Oligo-18. The recombination rate was represented in cM/Mb. In this study, recombination was observed in two intervals: the 119–535 interval between Oligo-pSc119.2–1 and Oligo-pTa535–1 signal sites and the 535–18 interval between Oligo-pTa535–1 and Oligo-18/Oligo-275.1 signal sites. The recombination rate and crossover interference were calculated according to the methods described by Ziolkowski et al. [27] using the following formulas: 119–535 cM = number of 5AL arms with recombination in 119–535 interval/total number of 5AL arms × 100, 535–18 cM = number of 5AL arms with recombination in 535–18 interval/total number of 5AL arms × 100, Recombination rate in 119–535 interval = 119–535 cM/48 Mb × 100, Recombination rate in 535–18 interval = 535–18 cM/59 Mb × 100, Observed double crossover (Obs DCO) = number of 5AL arms with recombination in both 119–535 and 535–18 intervals/total number of 5AL arms × 100, Expected double crossover (Exp DCO) = (119–535 cM/100) × (535–18 cM/100) × total number of 5AL arms, Coefficient of Coincidence (CoC) = Obs DCO/Exp DCO, and Crossover interference = 1 − CoC. Further, *t-*test and Chi-square test were carried out to determine significant differences in recombination rate and crossover interference, respectively, as described by Girard et al. [4]. An interference value close to zero indicates interference, and value close to 1, indicates no interference.

### Measurement of length between SC5A-479 and SC5A-527 probes on metaphase chromosomes

The length of metaphase 5AL segment (MSL) between the signals of the two single-copy probes SC5A-479 and SC5A-527 was measured. Four kinds of metaphase 5AL segments were measured: segments with Oligo-pSc119.2–1 signal (named 5AL^119^), segments with Oligo-pTa535–1 signal (named 5AL^535^), segments with signals of the two probes (named 5AL^119 + 535^), and segments without the signals of the two probes (named 5AL^No^). These four kinds of segments were derived from the four F_2_ generations, wheat cultivars CM36, CM90, XKM8, and KCM2. The length of the metaphase 5AL segment between SC5A-479 and SC5A-527 (MSL) was determined as relative metaphase length (RML) to avoid errors caused by chromosome condensation. RML = MSL/WL where WL represents the whole length of the 5AL arm from the central point of the centromere to the distal end. The MSL and WL were measured using cellSens Dimension software (Olympus Corporation, Tokyo, Japan). A one-way ANOVA was carried out using GraphPad Prism software (Version 5) for pairwise comparisons among the groups. Graphs were also plotted using GraphPad Prism software.

## Supplementary Information

**Additional file 1: Figure S1.** The recombination types of 5AL arms in the F_2_ progeny derived from CM39 × M1403. ‘5A-Rec1’ and ‘5A-Rec2’ indicate 5AL arms formed by recombination in the 535–18 interval. ‘5A-Rec3’ and ‘5A-Rec4’ indicate the 5AL arms formed by recombination in the 119–535 interval. ‘5A-DRec1’ and ‘5A-DRec2’ indicate the 5AL arms formed by recombination in both 535–18 and 119–535 intervals. Chromosomes were counterstained with DAPI (blue). Scale bar: 10 μm.

**Additional file 2: Figure S2.** The recombination types of 5AL arms in the F_2_ progeny derived from CM39 × MM43. ‘5A-Rec1’ and ‘5A-Rec2’ indicate 5AL arms formed by recombination in the 535–18 interval. ‘5A-Rec3’ and ‘5A-Rec4’ indicate the 5AL arms formed by recombination in the 119–535 interval. ‘5A-DRec1’ and ‘5A-DRec2’ indicate the 5AL arms formed by recombination in both 535–18 and 119–535 intervals. ‘5A’ indicates the 5A chromosome derived from CM39. Chromosomes were counterstained with DAPI (blue). Scale bar: 10 μm.

**Additional file 3: Figure S3.** The recombination types of 5AL arms in the F_2_ progeny derived from CM39 × XKM8 and 14 T105–1 × 14 T141–2. ‘5A-Rec3’ and ‘5A-Rec4’ indicate the 5AL arms formed by recombination in the 119–535 interval. ‘5A’ in (a) and (d) indicates the 5A chromosomes derived from XKM8 and 14 T141–2. ‘5A’ in (b) and (c) indicates the 5A chromosomes derived from CM39 and 14 T105–1. Chromosomes were counterstained with DAPI (blue). Scale bar: 10 μm.

**Additional file 4: Figure S4.** Sequential single-copy FISH and ND-FISH assays to determine the position of the signals of SC5A-479, SC5A-527, SC5A-586, Oligo-pSc119.2–1, Oligo-pTa535–1 and Oligo-18 probes. (**a, b, c**) Cells derived from CM39 × MM43 showing the signal sites of SC5A479 and SC5A527 close to those of Oligo-pSc119.2–1 and Oligo-pTa535–1, respectively, and 5AL^119^ and 5AL^535^ segments corresponding to the 119–535 interval between SC5A479 and SC5A527 signal sites. (**d, e, f**) Cells derived from CM39 × MM1403 showing 5AL^119 + 535^ and 5AL^No^ segments corresponding to the 119–535 interval between SC5A479 and SC5A527 signal sites. (**g, h, i**) Cells of KCM2 showing 5AL^119 + 535^ segment between SC5A479 and SC5A527 sites. (**j, k, l**) Cells derived from CM39 × MM43 showing the signal sites of Oligo-18 and SC5A-586 close to each other. Chromosomes were counterstained with DAPI (blue). Scale bar: 10 μm.

**Additional file 5: Table S1.** Oligonucleotide probes used for ND-FISH assays in this study.

## Data Availability

The materials used and/or analyzed in the current study are available from the corresponding author on reasonable request.

## Abbreviations

oligo: Oligonucleotide
ND-FISH: Non-denaturing fluorescence in situ hybridization
Obs DCO: Observed double crossover
Exp DCO: Expected double crossover
CoC: Coefficient of coincidence
MSL: Length of metaphase 5AL segment
RML: Relative metaphase length
WL: Whole length of 5AL arm
