# Supplementary figures and images for: FISH landmarks reflecting meiotic recombination and structural alterations of chromosomes in wheat (Triticum aestivum L.)
Source: BMC Plant Biol. 2021 Apr 6;21:167. doi: 10.1186/s12870-021-02947-1 (PMC8025513; doi:10.1186/s12870-021-02947-1)

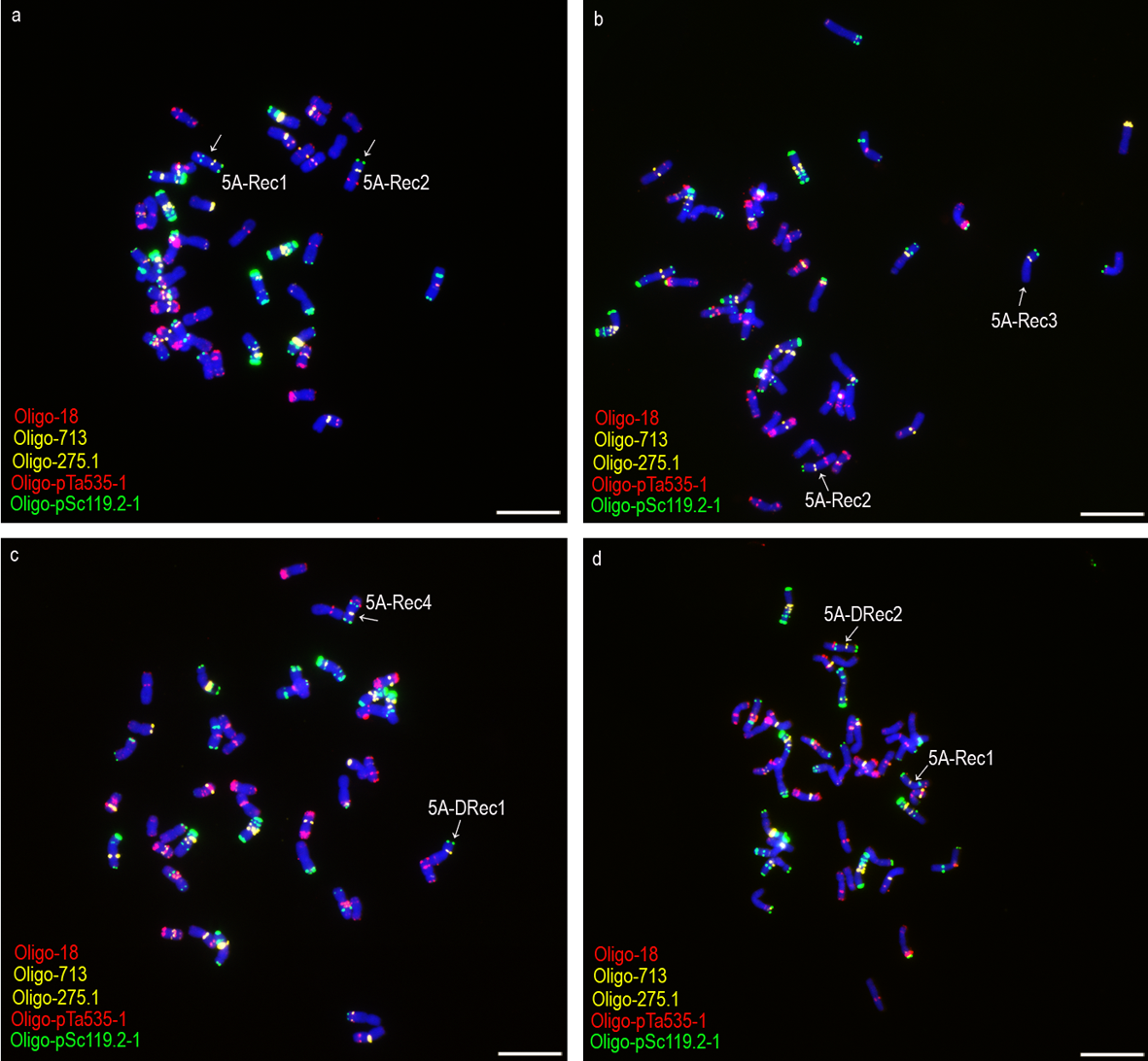

Supplement: Supplementary file 1 — Additional file 1: Figure S1. The recombination types of 5AL arms in the F2 progeny derived from CM39 × M1403. ‘5A-Rec1’ and ‘5A-Rec2’ indicate 5AL arms formed by recombination in the 535–18 interval. ‘5A-Rec3’ and ‘5A-Rec4’ indicate the 5AL arms formed by recombination in the 119–535 interval. ‘5A-DRec1’ and ‘5A-DRec2’ indicate the 5AL arms formed by recombination in both 535–18 and 119–535 intervals. Chromosomes were counterstained with DAPI (blue). Scale bar: 10 μm. [file 12870_2021_2947_MOESM1_ESM.tif]

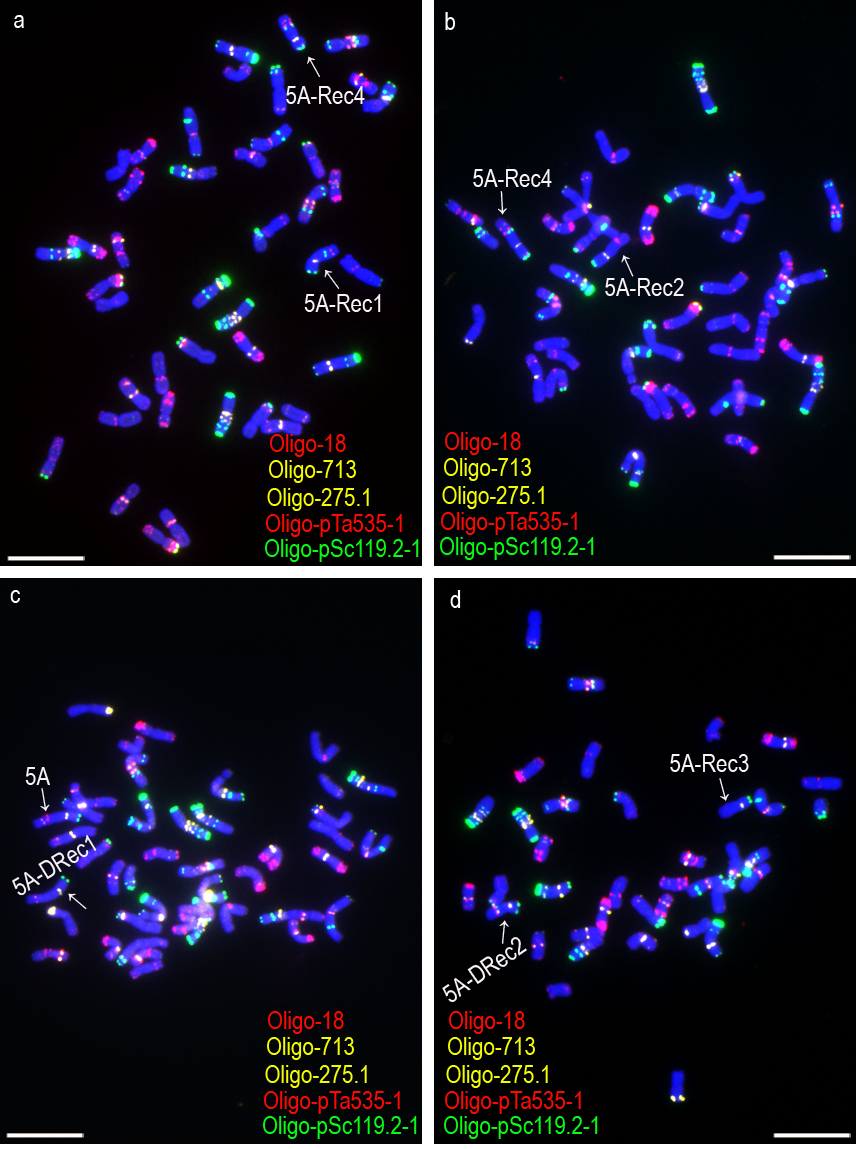

Supplement: Supplementary file 2 — Additional file 2: Figure S2. The recombination types of 5AL arms in the F2 progeny derived from CM39 × MM43. ‘5A-Rec1’ and ‘5A-Rec2’ indicate 5AL arms formed by recombination in the 535–18 interval. ‘5A-Rec3’ and ‘5A-Rec4’ indicate the 5AL arms formed by recombination in the 119–535 interval. ‘5A-DRec1’ and ‘5A-DRec2’ indicate the 5AL arms formed by recombination in both 535–18 and 119–535 intervals. ‘5A’ indicates the 5A chromosome derived from CM39. Chromosomes were counterstained with DAPI (blue). Scale bar: 10 μm. [file 12870_2021_2947_MOESM2_ESM.tif]

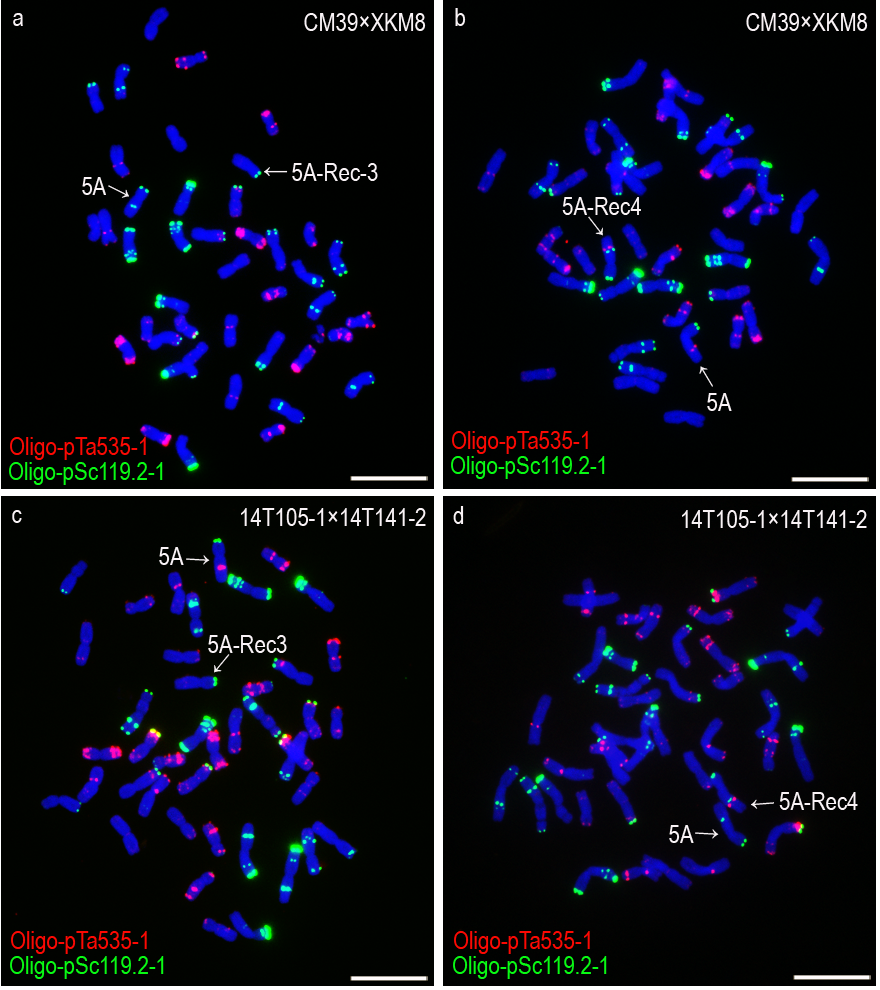

Supplement: Supplementary file 3 — Additional file 3: Figure S3. The recombination types of 5AL arms in the F2 progeny derived from CM39 × XKM8 and 14 T105–1 × 14 T141–2. ‘5A-Rec3’ and ‘5A-Rec4’ indicate the 5AL arms formed by recombination in the 119–535 interval. ‘5A’ in (a) and (d) indicates the 5A chromosomes derived from XKM8 and 14 T141–2. ‘5A’ in (b) and (c) indicates the 5A chromosomes derived from CM39 and 14 T105–1. Chromosomes were counterstained with DAPI (blue). Scale bar: 10 μm. [file 12870_2021_2947_MOESM3_ESM.tif]

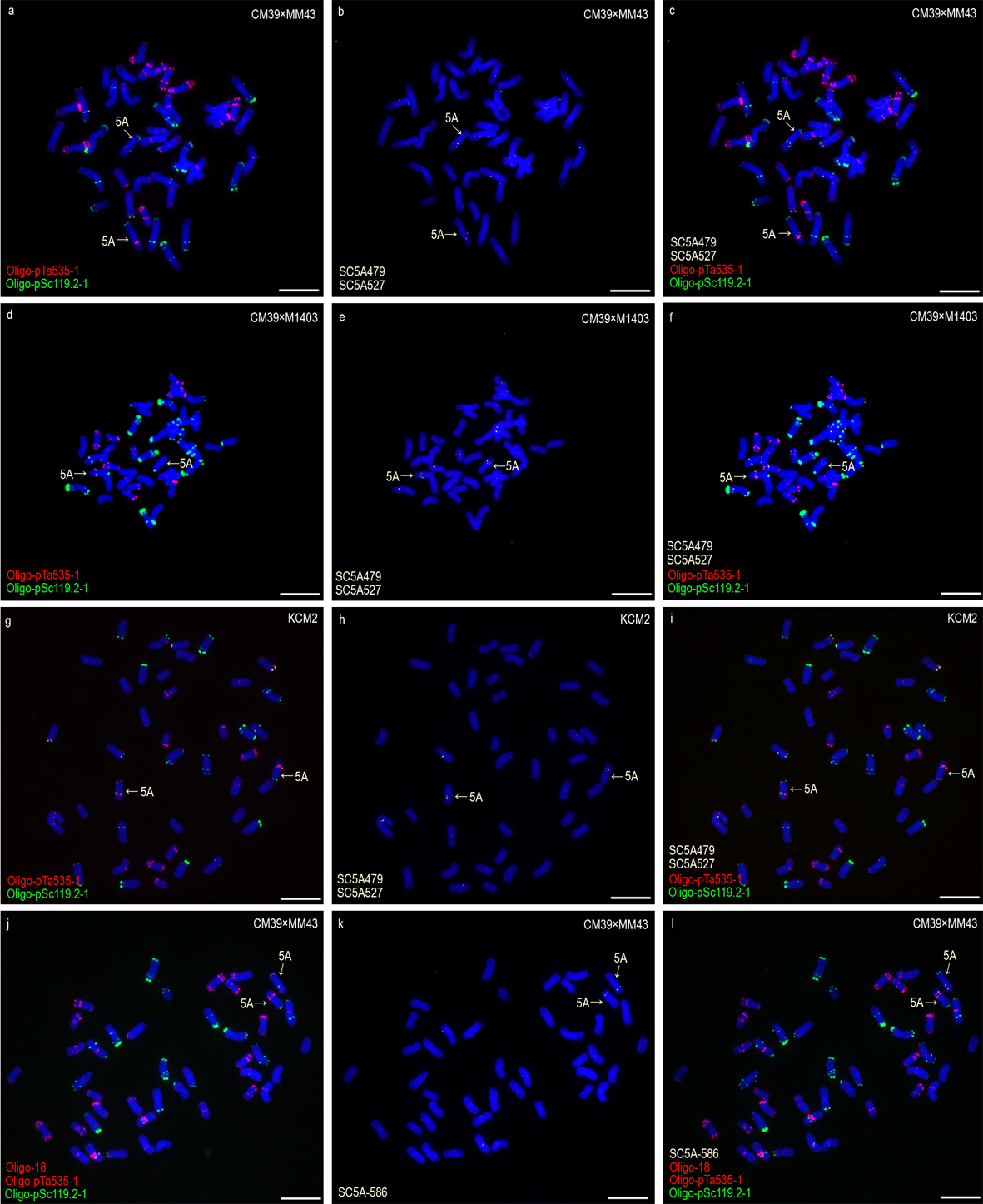

Supplement: Supplementary file 4 — Additional file 4: Figure S4. Sequential single-copy FISH and ND-FISH assays to determine the position of the signals of SC5A-479, SC5A-527, SC5A-586, Oligo-pSc119.2–1, Oligo-pTa535–1 and Oligo-18 probes. (a, b, c) Cells derived from CM39 × MM43 showing the signal sites of SC5A479 and SC5A527 close to those of Oligo-pSc119.2–1 and Oligo-pTa535–1, respectively, and 5AL119 and 5AL535 segments corresponding to the 119–535 interval between SC5A479 and SC5A527 signal sites. (d, e, f) Cells derived from CM39 × MM1403 showing 5AL119 + 535 and 5ALNo segments corresponding to the 119–535 interval between SC5A479 and SC5A527 signal sites. (g, h, i) Cells of KCM2 showing 5AL119 + 535 segment between SC5A479 and SC5A527 sites. (j, k, l) Cells derived from CM39 × MM43 showing the signal sites of Oligo-18 and SC5A-586 close to each other. Chromosomes were counterstained with DAPI (blue). Scale bar: 10 μm. [file 12870_2021_2947_MOESM4_ESM.tif]
